# A retrospective comparative study of anesthesia with remimazolam and remifentanil versus dexmedetomidine and remifentanil for transcatheter aortic valve replacement

**DOI:** 10.1038/s41598-023-43895-0

**Published:** 2023-10-10

**Authors:** Atsuhiro Kitaura, Shota Tsukimoto, Hiroatsu Sakamoto, Shinichi Hamasaki, Shinichi Nakao, Yasufumi Nakajima

**Affiliations:** Department of Anesthesiology, Kindai University Faculty of Medicine, 377-2 Ono-Higashi, Osakasayama, Osaka Japan

**Keywords:** Diseases, Medical research

## Abstract

Remimazolam, an ultrashort-acting benzodiazepine, allows for rapid and reliable arousal. Rapid awakening using remimazolam may be beneficial in transcatheter aortic valve replacement (TAVR), as it allows rapid detection of neurologic deficits. The purpose of this study was to compare arousal time and outcomes between monitored anesthesia care (MAC) with remimazolam and remifentanil and conventional MAC with dexmedetomidine, propofol, and remifentanil. This study was a single center retrospective study. All TAVR cases performed under MAC (MAC-TAVR) at our institution between 2019 and 2021 were included. Patients were classified by anesthesia method into remimazolam and dexmedetomidine groups. Among 258 MAC-TAVR patients, 253 were enrolled. After propensity score matching, 76 patients were assigned to each group. The time from end of drug-administration to arousal [20.0 (16.0, 24.0) min vs. 38.5 (30.0, 56.3) min, p < 0.0001] and the time from attempted-arousal to arousal [1.0 (1.0, 1.0) min vs. 12.5 (3.0, 26.8) min, p < 0.0001] were significantly shorter in the remimazolam group. There was no significant difference in the length of ICU stay [2.0 (2.0, 2.0) days vs. 2.0 (2.0, 2.0) days, p = 0.157] and postoperative hospital stay [6.0 (4.0, 9.0) days vs. 5.0 (4.0, 8.0) days, p = 0.262].

Trial registration: Clinical trial number: R03-123, Registry URL: https://center6.umin.ac.jp/cgi-open-bin/ctr/ctr_view.cgi?recptno=R000051635 Registration number: UMIN000045195, Principal investigator's name: Atsuhiro Kitaura, Date of registration: 20 August 2021.

## Introduction

Remimazolam is an ultra-short-acting benzodiazepine anesthetic that was launched in Japan in 2020^1^. The drug acts on the benzodiazepine binding site of the gamma-aminobutyric acid (GABA) A receptor and facilitates the action of GABA, a major inhibitory neurotransmitter, on the receptor, which is metabolized in the liver in a short period of time^2^. Compared to conventional agents, remimazolam is expected to further improve the quality of anesthesia as it has a short duration of action^3^, almost no cumulative effect^2,4^, minimal effect on circulatory dynamics^1^, and the presence of an antagonist (flumazenil). It is already being used for general anesthesia, but there is currently a lack of data on patients with specific complications.

Transcatheter aortic valve replacement (TAVR) is a treatment mainly performed in elderly patients with symptomatic severe aortic stenosis. Although it is much less invasive than conventional surgical aortic valve replacement, it carries a higher risk of cerebral embolism^5^.

Ideally, anesthesia for TAVR should provide rapid and clear awakening to ensure stable hemodynamics and early detection of cerebral embolisms. On the other hand, patients indicated for TAVR often suffer from circulatory fluctuations during induction of anesthesia due to aortic stenosis, delayed postoperative awakening due to advanced age, and cognitive decline^5^.

Both general anesthesia and local anesthesia with sedation were valid alternatives as the anesthetic strategies of TAVR. General anesthesia has the advantage in the early detection of complications through use of transesophageal echocardiography, easy to convert to open heart surgery in case of emergency. As devices have evoluted, the incidence of intraoperative complication has declined^6^. Therefore, TAVR using general anesthesia without tracheal intubation, called monitored anesthetic care (MAC), was initiated in Europe and the United States ahead of other countries, and its usefulness has been reported^7,8^.

Conventional MAC^9,10^ with dexmedetomidine and remifentanil has provided very stable anesthesia since it was introduced at our hospital. However, it must be used in combination with propofol, and skill is required by the anesthesiologist to determine the timing and dosage of the drugs. In addition, the long duration of action of dexmedetomidine and the lack of an antagonist may cause some patients to require several hours to awaken. On the other hand, the MAC method using mainly remimazolam, which was introduced in November 2020, seems to overcome the problems of conventional methods.

## Materials and methods

This study was a single center retrospective observational study, approved by the Institutional Review Board (IRB) of Kindai University Faculty of Medicine (no. R03-123). And we followed the opt-out model with approval of IRB of Kindai University Faculty of Medicine. The study was conducted in compliance with the Declaration of Helsinki. All patients who underwent transfemoral TAVR under monitored anesthesia care (MAC) in our hospital between January 2019 and December 2021 were retrospectively identified using the electronic medical records from our hospital without an a priori sample size calculation. Patients receiving preoperative sedation, intubation, beta stimulator, mechanical cardiopulmonary support, or maintenance of anesthesia with methods that did not fit the scope of this study were excluded. Patients with incomplete medical records were also excluded.

A total of 253 patients were eligible for this study, and their electronic medical records were reviewed. Data on demographics [age, sex, body mass index (BMI), New York Heart Association Functional Classification (NYHA)], comorbidities (hypertension, ischemic heart disease), preoperative surgical risk (Euro 2 score, single procedure non-CABG), severity of aortic valve stenosis [aortic valve area (AVA), mean velocity of aortic valve flow (AVPG mean)], left ventricular ejection fraction, anesthetic duration, intraoperative infusion volume, intraoperative blood gas analysis, and postoperative status were collected. The time required to awaken was used as the primary outcome. The secondary outcomes were days in ICU, postoperative hospital stay, anesthesia time, intraoperative fluid volume, lowest intraoperative potential of hydrogen (pH), and highest intraoperative arterial partial pressure of carbon dioxide (PaCO_2_).

### The definition of arousal time

Arousal was defined as a modified Aldrete score ≥ 9 points. Arousal time required was defined as the period of time between a point in time and arousal (modified Aldrete score ≥ 9 points). Two arousal time requirements were measured in this study: arousal time from the end of drug administration, which was measured from the end of sedative administration to arousal; and time required to awaken from the time of the attempted awakening (when the postoperative chest X-ray was confirmed). Rapidity of awakening in clinical anesthesia was defined as the time from the time of the awakening attempt, which was considered a more practical definition. The first assessment of modified aldolate score was done immediately after chest radiograph. The time measurement was started using a stopwatch, with the time at the start of the first evaluation set at 0 s. Immediately thereafter, flumazenil was administered only in the remimazolam group. During the stay in the operating room, the evaluation of arousal was constantly repeated. After entering the PACU, the nurse or anesthesiologist repeated the check every 5 min. The measurement start time and arousal time (When we first identified that the patient modified Aldrete score 9 or higher) were noted in the anesthesia record. If noradrenaline was used during the arousal evaluation, blood pressure assessment was reduced by 1 for convenience.

### Anesthesia methods

**Figure 1:**
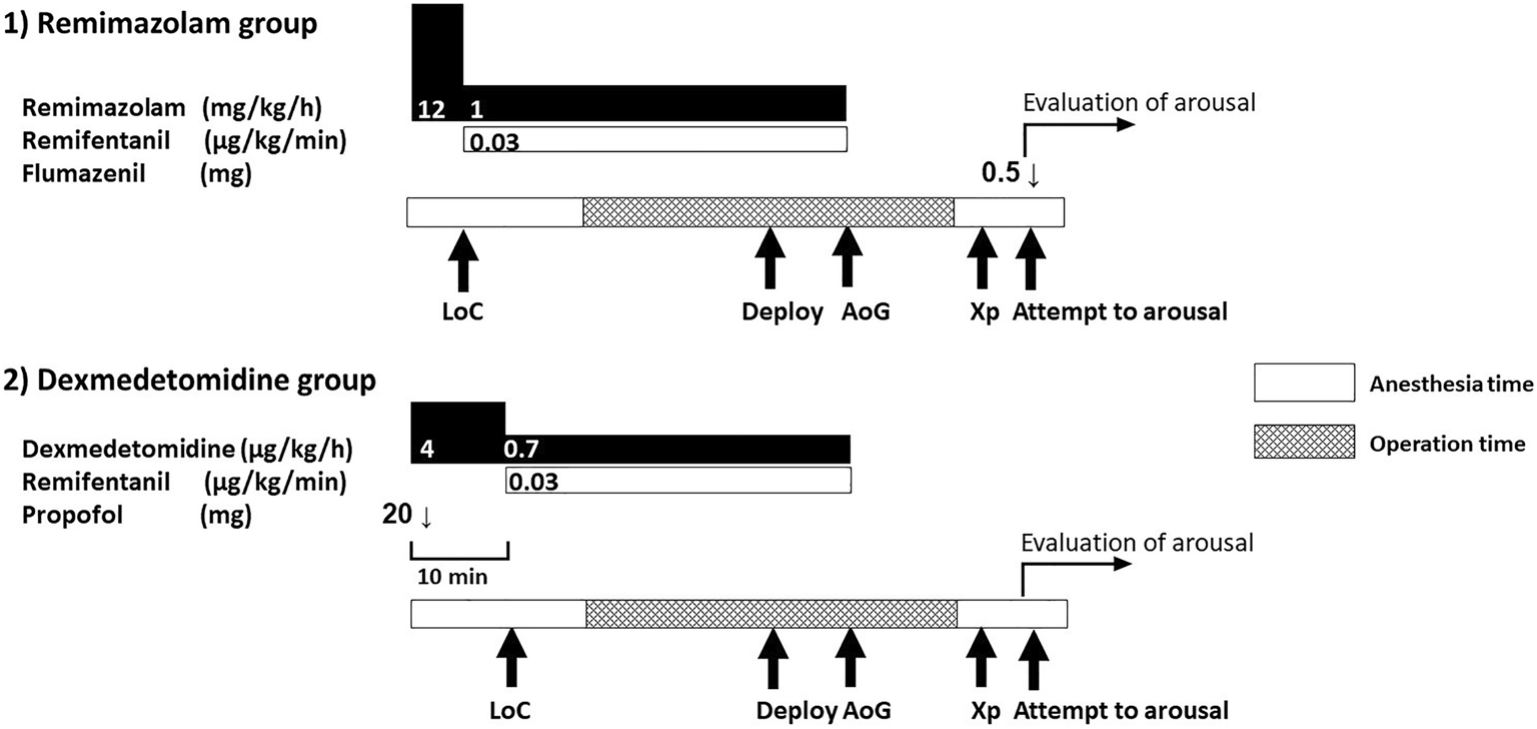
Anesthesia methods of the study. (1) The anesthesia method of remimazolam group, (2) the anesthesia method of dexmedetomidine group. *LoC* loss of consciousness, *Deploy* valve deployment, *AoG* confirmatory aortography after valve deployment, *Xp* chest X-ray.

We used our institution’s anesthetic protocol. No premedication was administered to patients undergoing TAVR. Angiotensin-converting enzyme inhibitors and angiotensin 2 receptor blockers and oral diabetes medications were discontinued on the day of surgery. After placing the patient on the operating table, devices for electrocardiography, pulse oximetry, noninvasive blood pressure monitoring, and bispectral index (BIS; Aspect Medical Systems, USA) were attached. Prior to the induction of anesthesia, an arterial blood gas analysis was performed under local anesthesia with an open arterial pressure line secured. MAC was maintained using the anesthesia method with remimazolam and remifentanil or the method with dexmedetomidine, propofol, and remifentanil. The choice of methods for maintaining anesthesia was decided based on the personal preference of the anesthesiologists. In the methods with remimazolam and remifentanil, remimazolam was administrated as a loading dose at 12 mg/kg/min and maintained at 1 mg/kg/h after loss of consciousness. In the methods with dexmedetomidine, propofol, and remifentanil, dexmedetomidine was administered as a loading dose at 4 μg/kg/h for 10 min, and maintained at 0.7 μg/kg/h. Furthermore, propofol was administered as a single 20 mg dose during the dexmedetomidine loading dose stage and then as a single dose as needed. When propofol was required was defined as during highly invasive procedures, such as urethral balloon insertion and rapid pacing; the appearance of body movements that interfere with the surgical procedure; and obvious signs of awakening. Propofol dosage and timing were determined by anesthesiologists. After loss of consciousness, a central venous catheter was secured in the right internal jugular vein under combined local anesthesia. At our institution, transthoracic echocardiography was used for intraoperative monitoring, and transesophageal echocardiography was not used. Respiration during anesthesia was managed with spontaneous breathing. The upper airway was secured using a Jaw elevation device (Hypnoz, USA). Intraoperative analgesia was administered by continuous infusion of 0.03 μg/kg/min remifentanil for both methods and 15 mg/kg acetaminophen was administered intravenously for postoperative analgesia for all cases. After completion of femoral artery cannulation, 100 units/kg of heparin was administered to prolong activated clotting time to at least 250 s. Dexamethasone sodium phosphate (6.6 mg) was administrated intravenously for all patients to prevent postoperative nausea and vomiting. Anesthetic depth was maintained in the range of 40–70 on the BIS monitor. Respiratory rate under spontaneous breathing was maintained at 10–25/min. In case of deviations, the remifentanil dose was adjusted. During anesthesia, noradrenaline was administered if the mean blood pressure fell below 65 mmHg. All anesthetics were discontinued upon completion of TAVR valve deployment. From this timing, the required time of arousal (from the end of administration) was measured. After surgery and postoperative x-rays, the patient's consciousness was checked. Measurement of time required to awaken was started at this time. Flumazenil 0.5 mg was administered through central venous line only in the remimazolam group immediately after the start of the measurement of time required to awaken. The patient was transferred to the Post-anesthesia Care Unit while continuing to confirm recovery of consciousness. The anesthesia method was illustrated in Fig. 1.

### Statistical analysis

All outcomes were compared between the remimazolam and dexmedetomidine groups using Wilcoxon signed-rank test. Propensity score matching was used to correct for selection bias and adjust for confounding factors. The factors included were age, sex, BMI, Euro Score 2, preoperative NYHA, preoperative AVA, AVPG mean, left ventricular ejection fraction, history of hypertension, and history of ischemic heart disease. First, propensity score matching was used to balance the covariates between the two groups. All patients were matched in a 1:1 ratio in order of nearest neighbor (Supplementary Digital Files 1). The caliper of nearest neighbor matching was 0.05. It was evaluated using absolute standardized difference (ASD), and matching was defined as a score of ≥ 0.1. Second, covariates with ASD values greater or equal to 0.1 were used as adjustment variables for the final model. ASD was used to evaluate the covariates and those with an ASD ≥ 0.1 were considered significant. SPSS statistics ver. 24.0 (IBM Japan, Ltd., Tokyo, Japan) was used for the analysis. Details regarding the propensity score regression model used in the present study are available in the Supplementary data (Supplementary Digital Files 2, 3 and 4). The post hoc test was used to estimate power. In the pre-matching sample had a mean difference of 30.4 in the required awakening time between the two groups, a standard deviation of 35.5 for all cases, an alpha error of 0.05, and a required sample size of 17 each in the parametric test. The minimum number of samples required for the nonparametric test should be increased by 15% from the required number of samples for the parametric test^11^. The minimum number of samples required is therefore 20 for each. Therefore, the sample size of our study was deemed adequate. In addition, our sample size was also considered adequate in reference to the paper on sample size for studies using propensity score matching^12^.

### Ethical approval of study

This study was approved by the IRB of Kindai University Faculty of Medicine (no. R03-123), and we followed the opt-out model with approval of IRB of Kindai University Faculty of Medicine. The IRB of Kindai University Faculty of Medicine waived the need to obtain informed consent from study participants in this study. Our study is also registered in University hospital medical information network (UMIN), a clinical research database in Japan. This study also conformed to Japanese law. This study was conducted in compliance with the Declaration of Helsinki.

## Results

**Figure 2:**
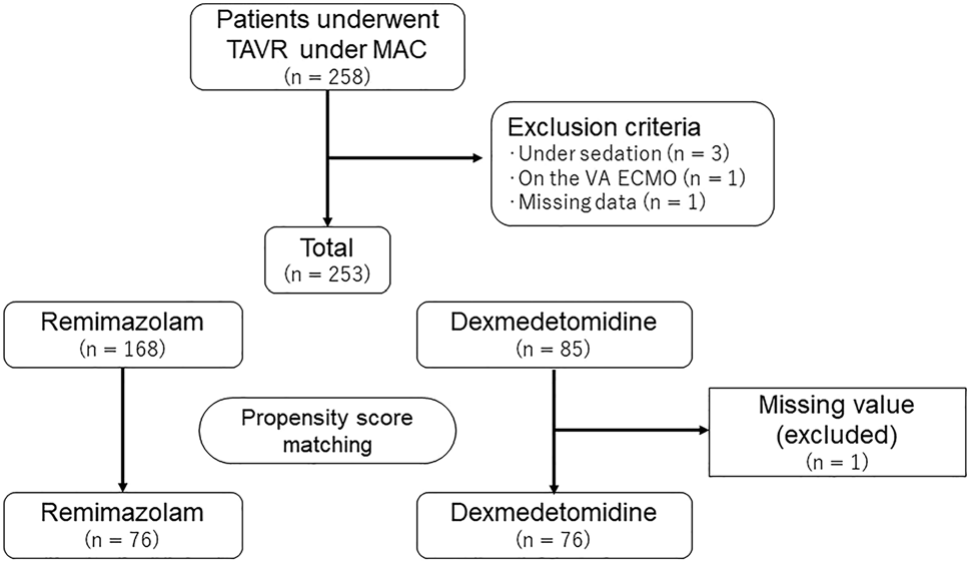
Flow chart of the study. *TAVR* transcatheter aortic valve replacement, *MAC* monitored anesthesia care.

Among 258 patients with TAVR who underwent surgery under MAC, 253 were enrolled in the analysis (Fig. 2), which consisted of 168 (66%) patients who underwent MAC with remimazolam protocol and 85 (34%) patients who received MAC with dexmedetomidine protocol. During propensity score matching, one patient was excluded because of missing data; thus, 252 were included in the matching. After propensity score matching, 76 patients were assigned to the remimazolam group and 76 patients to the dexmedetomidine group (Fig. 2). All patients were performed TAVR by transfemoral approach. The type of valve used were Sapien 3 (Edwards Lifesciences Co., USA) and Evolute series (Medtronic plc, Ireland). Remimazoram group included 54 Sapien cases and 22 Evolut cases. Fourty-three Sapien cases and 33 Evolute cases were included in dexmedetomidine group.

Table 1 shows the characteristics of the patients before and after propensity score matching. Age, BMI, NYHA, Euro 2 score, AVA, AVPG mean, and a history of ischemic heart disease were significantly different between the groups before matching. ASA physical status (ASA-PS) was not considered in the matching process, because all patients who were enrolled in this study had a grade of 4. The ASD of factors including age, sex, BMI, Euro Score 2, preoperative NYHA, preoperative AVA, AVPG mean, left ventricular ejection fraction, history of hypertension, and history of ischemic heart disease were calculated. After matching, all covariates had an ASD < 0.1 in all 152 patients.

**Table 1:** Patient characteristics before and after propensity score matching.

	Before propensity score matching (n = 253)						After propensity score matching (n = 152)				
	n	Remimazolam group	n	Dexmedetomidine group	P-value	ASD	n	Remimazolam group	n	Dexmedetomidine group	ASD
Gender	168		85		0.764		76		76		
Male		45, 26.8		21, 24.7		4.8%		17,22.4		17,22.4	0.0%
Female		123, 73.2		64, 75.3		4.8%		59,77.6		59,77.6	0.0%
Age	168	83.4 ± 6.0	85	84.7 ± 5.1	0.087	23.5%	76	84.2 ± 5.8	76	84.5 ± 5.1	5.5%
BMI	168	22.4 ± 3.5	85	21.6 ± 4.1	0.094	21.8%	76	21.8 ± 3.3	76	21.7 ± 4.0	3.0%
NYHA	168		85		0.019		76		76		
1		10, 6.0		15, 17.6		36.9%		10,13.2		8,10.5	8.2%
2		128, 76.2		54, 63.5		27.9%		50,65.8		53,69.7	8.5%
3		27, 16.1		13, 15.3		2.1%		14,18.4		13,17.1	3.4%
4		3, 1.8		3, 3.5		10.9%		2,2.6		2,2.6	0.0%
Euro 2 score	168	3.5 ± 3.0	85	4.3 ± 5.9	0.147	17.3%	76	3.7 ± 3.2	76	4.0 ± 4.9	7.9%
AVA	167	0.76 ± 0.22	84	0.70 ± 0.18	0.041	28.5%	76	0.73 ± 0.19	76	0.71 ± 0.18	8.8%
AVPG mean	164	42.6 ± 17.2	84	44.2 ± 15.3	0.457	10.2%	76	42.8 ± 16.6	76	43.6 ± 15.0	5.1%
LVEF	167	0.63 ± 0.11	85	0.64 ± 0.12	0.760	4.0%	76	0.64 ± 0.11	76	0.64 ± 0.12	4.7%
The history of hypertension	168	158, 94.0	85	78, 91.8	0.596	8.9%	76	69 , 90.8	76	69,90.8	0.0%
The history of ischemic heart disease	168	48, 28.6	85	30, 35.3	0.314	14.5%	76	25 , 32.9	76	27,35.5	5.5%

Values are presented as n, %, median ± sd.

*BMI* body mass index, *NYHA* NewYork Heart Assiciation Classification, *AVA* aortic valve area, *AVPG mean* mean pressure gradient across the aortic valve, *LVEF* left ventricle ejection fraction, *ASD* absolute standardized difference.

Variables which were shown "a" were analized using Fisher's exact test. And variables which were marked "b" were analized using unpaired t test.

Propensity score matching was performed using the algorithm of nearest neighbor matching. Caliper width: 0.05. Sampling: no replacement. Composition ratio: one-to-one pair matching.

*ASD* absolute standardized difference. ASD < 10% was determined to be in balance.

Table 2 shows the time required to awaken and the number of days in the ICU, postoperative hospital stay, anesthesia time, intraoperative fluid volume, lowest intraoperative arterial pH, and highest intraoperative PaCO_2_. Time from the end of drug administration to awakening was significantly shorter in the remimazolam group (20.0 [16.0, 24.0] min vs. 38.5 [30.0, 56.3] min, p < 0.0001). Time from attempted arousal to arousal was significantly shorter in the remimazolam group (1.0 [1.0, 1.0] min vs. 12.5 [3.0, 26.8] min, p < 0.0001). In particular, the remimazolam group had a very short turnaround time of about 1 min. There is no significant difference of the outcomes of TAVR procedure. Details include, the success rate of TAVR was 100% in both groups. There were two cases of moderate postoperative aortic regurgitation due to annulus severe calcification in each group. There were two bleeding complications in the remimazolam group and one in the dexmedetomidine group. In one case in the remimazolam group, the hemostatic device (Perclose ProGlide™, Abbott, USA), did not work due to poor vascularity of the puncture site. The other two cases were due to inexperienced hemostatic maneuvers. There was no case who diagnosed perioperative brain stroke. There was no significant difference in postoperative course such as ICU stay (2.0 [2.0, 2.0] days vs. 2.0 [2.0, 2.0] days, p = 0.157) and postoperative hospital stay (6.0 [4.0, 9.0] days vs. 5.0 [4.0, 8.0] days, p = 0.262). Moreover, there were no significant differences in anesthesia time (91.5 [77.0, 108.3] min vs. 96.0 [80.5, 110.8] min, p = 0.929), intraoperative fluid volume (1100.0 [812.5, 1550.0] mL vs. 1200.0 [900.0, 1675.0] mL, p = 0.201) and intraoperative remifentanil use (66.3 [51.0, 82.0] µg vs. 67.4 [51.6, 81.6] µg, p = 0.486). The highest intraoperative PaCO_2_ values were significantly higher in the remimazolam group (51.7 [42.6, 56.6] mmHg vs. 45.3 [40.5, 49.8] mmHg, p = 0.002). The lowest intraoperative pH was significantly lower in the remimazolam group (7.31 [7.3, 7.4] vs. 7.33 [7.3, 7.4], p < 0.001). No cases required airway clearance with supraglottic devices or mechanical ventilation. Intraoperative noradrenaline use was higher in the remimazolam group (0.17 [0.01, 0.25] mg vs. 0.08 [0.0, 0.11] mg, p = 0.002). The number of cases who require noradrenaline was 59 (78%) in the remimazolam group and 54 (72%) in the dexmedetomidine group. The dose of propofol used was 77.3 ± 47.3 mg (mean ± SD) in the dexmedetomidine group; however, no patients in the remimazolam group were administered the drug.

**Table 2:** Comparison of outcomes between remimazolam and DEX groups after propensity score matching.

Variables	n	Remimazolam group	n	DEX group	P-value
Arousal time (from the end of anesthetic administration) (minutes)	76	20 [16.0, 24.0]	76	38.5 [30.0, 56.3]	< 0.001
Arousal time (from the attempted awaking) (minutes)	76	1 [1.0, 1.0]	76	12.5 [3.0, 26.8]	0
Anesthesia time (minutes)	76	91.5 [77.0, 108.3]	76	96 [80.5, 110.8]	0.929
Infusion volume (mL)	76	1100 [812.5, 1550.0]	76	1200 [900.0, 1675.0]	0.201
Intraoperative remifentanil dose (µg)	76	66.3 [51.0, 82.0]	76	67.4 [51.6, 81,6]	0.486
Intraoperative noradrenaline dose (mg)	76	0.17 [0.01, 0.25]	76	0.08 [0.0, 0.11]	0.002
Cases requiring noradrenaline (n, %)	76	59 (78%)	76	54 (72%)	
Intraoperative minimum value of Ph	76	7.3 [7.3, 7.4]	76	7.3 [7.3, 7.4]	0.002
Intraoperative maximum value of PaCO_2_ (Torr)	76	51.7 [42.6, 56.6]	76	45.3 [40.5, 49.8]	< 0.001
ICU stay (days)	76	2 [2.0, 2.0]	76	2 [2.0, 2.0]	0.157
Postoperative hospitalization days (days)	76	6 [4.0, 9.0]	76	5 [4.0, 8.0]	0.262

Data are presented as median [IQR].

P-value: Wilcoxon signed-rank test.

*DEX* dexmedetomidine, *PaCO*_*2*_ partial arterial pressure of carbon dioxide, *ICU* intensive care unit, *IQR* interquartile range.

## Discussion

The remimazolam group had significantly faster arousal than the dexmedetomidine group. Remimazolam is a newer, ultra-short-acting benzodiazepine sedative^1^. Remimazolam is rapidly metabolized in the liver^3^ and has virtually no cumulative effects^2,4^. Remimazolam has a half-life time of approximately 46 min. On the other hand, dexmedetomidine, the most commonly used drug for MAC, has a half-life of approximately 2 h^13^. As remimazolam is short-acting, it may have benefits in the treatment of TAVR patients who are an older and more frail patient population. The half-life of remimazolam is comparable to that of its antagonist, flumazenil, at approximately 47 min. In the present study, antagonism with flumazenil was highly reliable, with no cases of delayed arousal. TAVR tends to be performed in older patients with higher frailty and a higher risk of anesthetic complications, as they tend to be more susceptible to complications^14^. Remimazolam has already been reported to be safe in high-risk patients with ASA-PS3^15^, but these results suggest that remimazolam may be safe in patients with ASA-PS4 who have severe aortic stenosis. TAVR is a high-risk procedure with cerebral embolic complications of about 5%^5^. Therefore, prompt and reliable awakening is considered very important for early detection of cranial nerve complications. The effects of sedation have already been reported as a cause of delayed stroke detection^16^. In addition, remimazolam is reported to be advantageous for postoperative recovery^17^. Clear awaking may also reduce the workload of the physician in charge by providing an opportunity to rule out cranial nerve complications immediately after surgery, which may simplify postoperative patient explanation and patient management.

On the other hand, intraoperative respiratory depression was stronger in the remimazolam group than in the dexmedetomidine group. As a result, pH was significantly lower in the remimazolam group. In addition, PaCO_2_ was significantly higher in the remimazolam group. Noradrenaline use was also significantly higher in the remimazolam group, and circulatory depression was stronger in the remimazolam group. This result may not dispel concerns about the occurrence of severe hypotension with remimazolam in TAVR, as a report highlighted the possibility of sustained hypotension even with dexmedetomidine^18^. However, there were no cases of conversion to general anesthesia due to respiratory arrest or circulatory collapse, and noradrenaline use was relatively low. Therefore, we believe that respiratory depression and increased use of noradrenaline were within the range that could have been easily managed by a typical clinical anesthesiologist. On the other hand, it is possible that the use of remimazolam may have been excessive in this study due to the influence of the protocol set up with the dosage as per the package insert. Recently, remimazolam doses have been reduced in many institutions. A reduction of the loading dose rate to 6 mg/kg/h has been reported to reduce hypotension^1^. It has also been reported that maintenance doses can be reduced to 0.3–0.6 mg/kg/h, using BIS as an indicator^4,19,20^. Thus, more flexible patient-specific adjustments may further reduce the respiratory and circulatory depression observed in this study. Our findings suggest that there were no adverse effects on prognosis at this dose, as there was no difference between the remimazolam group and the dexmedetomidine group in terms of ICU stay and postoperative hospital stay.

This study had several limitations. First, this study had a retrospective design. There were limited data items available and selection bias attributable to anesthesiologists could not be excluded as they decided between administering remimazolam or dexmedetomidine for MAC. Propensity score matching was performed to adjust for confounding factors; however, due to sample size, only 10 variables were used for matching (age, sex, BMI, NYHA, Euro 2 score, AVA, AVPG mean, LVEF, history of hypertension, and history of ischemic heart disease). Second, this was a small, single-center study. The results could be different in settings at other facilities. It should be noted that the facility where this study was conducted was a multi-center facility for sedated TAVR and the quality of the surgeons and other team members was assured. Although the sample size in this study was adequate for statistical power, larger studies are necessary to confirm our findings.

In conclusion, this study showed that MAC with remimazolam and remifentanil provides rapid and reliable arousal in TAVR without having a negative impact on patient prognosis.

### Supplementary Information

Supplementary Information 1.

Supplementary Information 2.

Supplementary Information 3.

Supplementary Information 4.

## Data Availability

The datasets generated during and/or analysed during the current study are available from the corresponding author on reasonable request.
