# Supplementary material for: A retrospective comparative study of anesthesia with remimazolam and remifentanil versus dexmedetomidine and remifentanil for transcatheter aortic valve replacement
Source: Sci Rep. 2023 Oct 10;13:17074. doi: 10.1038/s41598-023-43895-0 (PMC10564871; doi:10.1038/s41598-023-43895-0)
Supplement: Supplementary file 2 — Supplementary Information 2. [file 41598_2023_43895_MOESM2_ESM.pdf]

Supplement 2. Evaluation of Propensity Score (C statistic)

C statistic	95% CI	P-value
0.649	0.574 , 0.723	< 0.001

Propensity score calculation variables: sex, age, body mass index, New York Heart Association Classification, Euro 2 Score, aortic valve area, mean pressure gradient across the aortic valve, left ventricle ejection fraction, history of hypertension, history of ischemic heart disease
