# Supplementary figures and images for: A retrospective comparative study of anesthesia with remimazolam and remifentanil versus dexmedetomidine and remifentanil for transcatheter aortic valve replacement
Source: Sci Rep. 2023 Oct 10;13:17074. doi: 10.1038/s41598-023-43895-0 (PMC10564871; doi:10.1038/s41598-023-43895-0)

Supplement 3. ROC Curve of C statistic

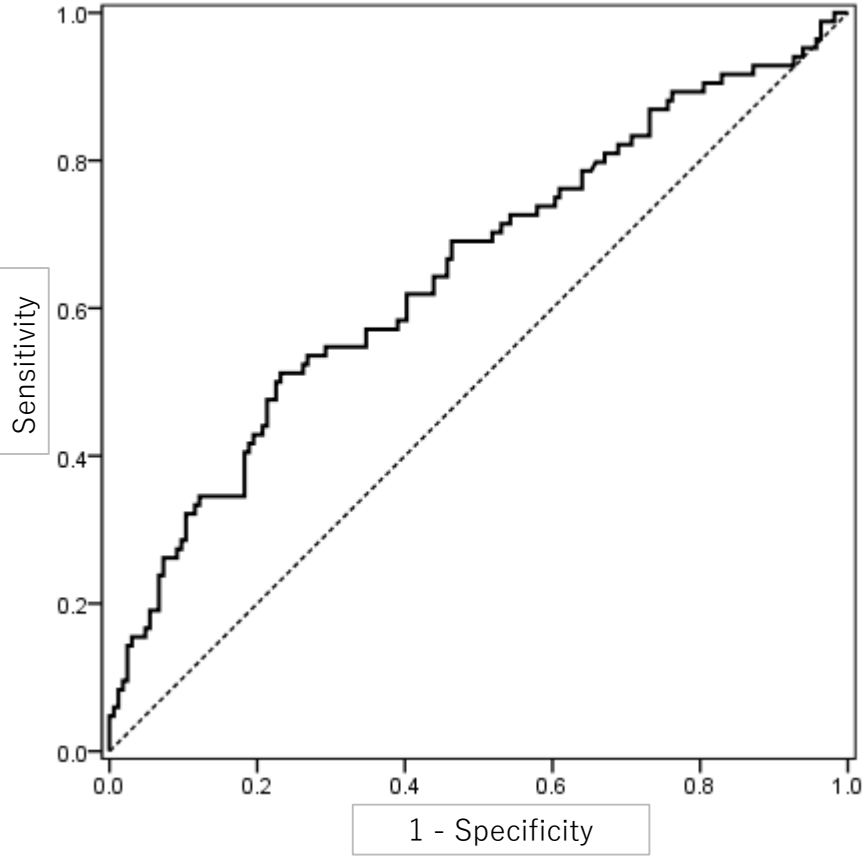

Supplement: Supplementary file 3 — Supplementary Information 3. [file 41598_2023_43895_MOESM3_ESM.pdf]

Supplement 4. ROC Curve of C statistic

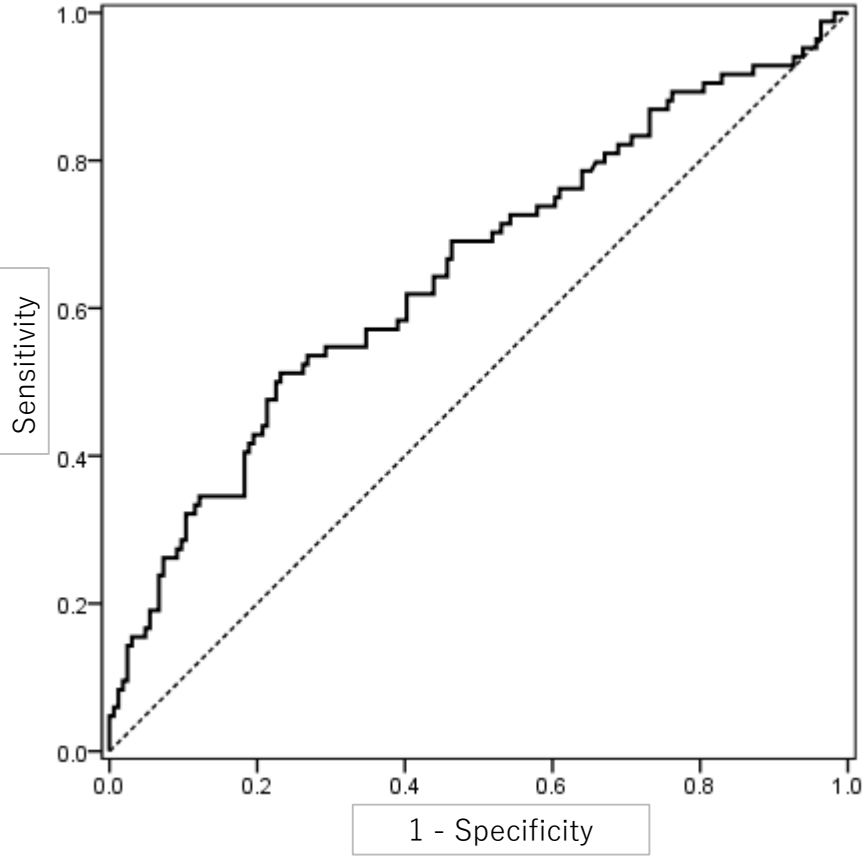

Supplement: Supplementary file 4 — Supplementary Information 4. [file 41598_2023_43895_MOESM4_ESM.pdf]
